# Carbonic anhydrase IX in oligodendroglial brain tumors

**DOI:** 10.1186/1471-2407-8-1

**Published:** 2008-01-04

**Authors:** Sally Järvelä, Seppo Parkkila, Helena Bragge, Marketta Kähkönen, Anna-Kaisa Parkkila, Ylermi Soini, Silvia Pastorekova, Jaromir Pastorek, Hannu Haapasalo

**Affiliations:** 1 Department of Pathology, Centre for Laboratory Medicine, Tampere University Hospital, Tampere, Finland; 2 Institute of Medical Technology, University of Tampere and Tampere University Hospital, Tampere, Finland; 3 Department of Genetics, Centre for Laboratory Medicine, Tampere University Hospital, Tampere, Finland; 4 Department of Neurology, Tampere University Hospital, Tampere, Finland; 5 Department of Pathology, University of Oulu, Oulu, Finland; 6 Centre of Molecular Medicine, Institute of Virology, Slovak Academy of Sciences, Slovak Republic

## Abstract

**Background:**

Carbonic anhydrase IX is a hypoxia-induced enzyme that has many biologically important functions, including its role in cell adhesion and invasion.

**Methods:**

This study was set out to investigate the role of CA IX in a series of 86 oligodendroglial brain tumors (71 primary and 15 recurrent; 48 pure oligodendrogliomas and 40 mixed oligoastrocytomas).

**Results:**

80% of the tumors showed CA IX expression by immunohistochemistry. Tumors with moderate or strong CA IX expression had decreased level of cell proliferation compared to weak or no CA IX expression (median 2.9 vs. 5.8, p = 0.015). CA IX correlated with two antioxidative enzymes, manganese superoxide dismutase (MnSOD) and regulatory gammaglutamylcysteine synthetase (GLCL-R): CA IX expression was significantly higher in MnSOD-positive tumors (p = 0.008) and decreased in GLCL-R-positive tumors (p = 0.044). In Cox multivariate analysis CA IX expression, patient age and histological component (pure oligodendroglioma vs. mixed oligoastrocytoma) showed independent prognostic values (p = 0.009, p = 0.003 and p = 0.022, respectively), CA IX positivity predicting poorer outcome.

**Conclusion:**

CA IX was proved to be an independent prognostic indicator in oligodendroglial brain tumors, and it also correlates reversely with cell proliferation. It may have a role in the biology of oligodendrogliomas, and most interestingly, as it is mainly expressed in tumor tissue, CA IX could serve as a target molecule for anticancer treatments.

## Background

Oligodendrogliomas account for approximately 5–18% of all intracranial gliomas and they occur primarily in the frontal or temporal lobes of the cerebrum [1,2]. It has been suggested that oligodendrogliomas were previously underdiagnosed, and there are several novel studies where their incidence is considerably higher, up to 33% of gliomas [3]. Accurate diagnosis is important in the case of oligodendrogliomas because the pathophysiology, treatment options and prognosis vary from that of diffusely infiltrating astrocytomas[1].

There are at least two different genetic pathways for the tumorigenesis of oligodendrogliomas. Loss of chromosomal regions on 1p and 19q is characteristic of oligodendrogliomas, but there is also evidence for another subset of oligodendrogliomas that have amplification of *EGFR *oncogene, loss of heterozygosity on chromosome 10 and homozygous deletion of the *CDKN2A *tumor suppressor gene. This seems to exclude the more common genetic changes in 1p and 19q [4,5].

Antioxidant enzymes and related proteins (AOEs) are part of the cellular protection mechanisms against functional and structural damage caused by reactive oxygen species [6]. Increased oxidant stress and/or diminished levels of AOEs lead to multiple injurious consequences in living cells, including susceptibility to genetic alterations and carcinogenesis. AOEs such as manganese superoxide dismutase (MnSOD), glutathione associated enzymes (GLCL-C and GLCL-R) and thioredoxin-thioredoxin reductase (Trx, TrxR) have been shown to correlate with tumor grade, metastasis and poor prognosis in invasive carcinomas such as lung and gastrointestinal malignancies and we have earlier studied them in oligodendroglial brain tumors [7-10].

Carbonic anhydrase IX (CA IX) is a tumor-associated metalloenzyme that belongs to the physiologically important family of at least 13 different mammalian carbonic anhydrases, CAs [11-13]. It is localized in the plasma membrane and like other enzymatically active CA isoenzymes (CA I-IV, VA, VB, VI, VII, XII-XV), it contains four important histidine residues: three residues for the coordination of a zinc ion in the active site and one for a proton shuttle. CAs catalyze the reversible interconversion between CO_2 _and HCO_3 _^-^, one of the most fundamental chemical reactions in cells. This reaction is essential for organisms as it influences respiration, pH regulation and homeostasis, exchange of electrolytes and several metabolic biosynthetic pathways [12-15]. Aberrant changes in this fine machinery are implicated in many diseases, including cancer.

CA IX has a special role among human CA isoenzymes because it can only be found in few normal tissues, but it is abundant in several tumors, such as colorectal, bladder, cervical, lung and breast carcinomas [16-18]. Even though the expression of CA IX in these carcinomas is evident, the tissues from which the carcinomas are originally derived are known to be CA IX-negative or they show only low enzyme expression. Furthermore, the few normal tissues or cell types that express CA IX, such as gastrointestinal and gallbladder epithelial cells, have been reported to lose the expression of CA IX during carcinogenesis [19-22]. This quite exceptional phenomenon makes CA IX an interesting tumor-associated protein.

CA IX expression is strongly induced by hypoxia. This transcriptional activation is accomplished via the HIF-1 transcription factor, which accumulates in tissue under the hypoxic condition that is often present in growing tumors. That, in turn, is an outcome of poorly organized and insufficient vasculature in uncontrollably growing malignant tissue. The HIF-1 transcription factor is a trigger for several hypoxia-regulated genes linked to cell survival, proliferation, apoptosis, angiogenesis and metabolism in tumor cells. The activation of these genes helps the cell to adapt to the stress caused by low oxygen level in a particular tissue. Earlier studies have shown that CA IX expression is induced as early as two hours after HIF activation and persists for several days, even if HIF-1 expression has ceased. This means that CA IX reflects both previous and present hypoxia in cells [23-25]. In addition to the important correlation between hypoxia and CA IX expression, it has been found that CA IX expression correlates with poor prognosis in various tumors [26-29]. It has also been suggested that CA IX has a direct role in tumor progression and the regulation of pH balance during tumorigenesis. CA IX has effects on cell adhesion, too, and it has been suggested to play a role in tumor invasion through weakening of cell-cell adhesion as E-cadherin is competing for β-catenin [30]. However, the effect of CA IX on tumor cell invasion is still under debate as some recent results have suggested that there is no evidence of such a correlation [31]. Even though we do not know the exact mechanisms of CA IX in cancer development, it is undoubtedly a promising target for anticancer treatments. It is also known that CA IX is strongly inhibited by aromatic and heterocyclic sulfonamides, and several other potent inhibitors for CAs have already been designed [32,33]. These facts prompted us to investigate the expression of CA IX in a series of histopathologically and genetically verified oligodendroglial brain tumors.

## Methods

### Patients

We analyzed oligodendroglial tumor samples from 86 patients who underwent surgery during 1980–2004 at Tampere University Hospital, Turku University Hospital and Kuopio University Hospital, Finland. 71 patients had a primary tumor and 15 a recurrent tumor. Of these tumors 55 were grade II and 31 were grade III. Forty-eight tumors were pure oligodendrogliomas and 38 were oligodendrogliomas with an additional astrocytic component (mixed oligoastrocytomas). All the patients underwent neurosurgical operation with the intention of gross radical tumor resection. The median age of the patients at the time of operation was 40.0 years (mean 41.5, SD +14.2). The median follow-up time was 3.5 years (mean 4.9, SD 4.5). During the follow-up, 43 patients died.

### Tumor samples

The tumor samples were fixed in 4% phosphate-buffered formaldehyde and embedded in paraffin. Paraffin sections were stained with hematoxylin and eosin. Histopathological typing and grading were carried out according to WHO criteria (1) independently by two experienced neuropathologists. Histologically representative and most proliferative oligodendroglial tumor regions were selected by a neuropathologist (HH) and the samples from these areas were placed in tissue microarray blocks using a custom-built instrument (Beecher Instruments, Silver Spring, MD, USA). The diameter of the tissue cores in the microarray block was 600 μm. The tumors in the microarray analysis comprised both pure oligodendrogliomas and mixed oligoastrocytomas (for details, see Table 1.).

**Table 1:** The expression of carbonic anhydrase IX in oligodendrogliomas.

**CA IX expression N(%)**	**Negative**	**Weak**	**Moderate**	**Strong**	**Total (N)**
**All tumors**	17 (20%)	53 (62%)	9 (10%)	7 (8%)	86
**Primary tumors**	14 (20%)	46 (65%)	7 (10%)	4 (5%)	71
**Recurrent tumors**	3 (20%)	7 (47%)	2 (13%)	3 (20%)	15
**Pure oligodendrogliomas**	10 (21%)	30 (63%)	4 (8%)	4 (8%)	48
**Mixed oligoastrocytomas**	7 (18%)	23 (61%)	5 (13%)	3 (8%)	38
**Grade II**	12 (22%)	31 (56%)	7 (13%)	5 (9%)	55
**Grade III**	5 (16%)	22 (71%)	2 (6,5%)	2 (6,5%)	31

### Immunohistochemical procedures

#### CA IX

The monoclonal antibody M75, which recognizes the N-terminal proteoglycan domain of human CA IX, has been described previously [34].

Automated immunostaining, which was performed using Power Vision+™ Poly-HRP IHC Kit (ImmunoVision Technologies, Co.) reagents, included the following steps: (a) rinsing in wash buffer; (b) treatment in 3% H_2_O_2 _in ddH_2_O for 5 min and rinsing in wash buffer; (c) blocking with Universal IHC Blocking/Diluent for 30 min and rinsing in wash buffer; (d) incubation with the primary antibody (M75 for CA IX) or normal rabbit serum (NRS) diluted 1:200 (M75) or 1:2000 (NRS) in Universal IHC Blocking/Diluent for 30 min; (e) rinsing in wash buffer for 3 × 5 min; (f) incubation in poly-HRP-conjugated anti-rabbit or anti-mouse IgG for 30 min and rinsing in wash buffer for 3 × 5 min; (g) incubation in DAB (3,3'-diaminobenzidine tetrahydrochloride) solution (one drop of DAB solution A and one drop of DAB solution B in 1 ml) ddH_2_O for 6 min; (h) rinsing with ddH_2_O; (i) CuSO_4 _treatment for 5 min to enhance the signal; and (j) rinsing with ddH_2_O. All procedures were carried out at room temperature. The sections were mounted in Entellan Neu (Merck; Darmstadt, Germany) and finally examined and photographed with a Zeiss Axioskop 40 microscope (Carl Zeiss; Göttingen, Germany).

#### Antioxidative enzymes

The antibodies for the AOEs were as follows: a polyclonal rabbit anti-human antibody to MnSOD (a gift from Professor J.D. Crapo, National Jewish Medical Center, Denver, CO, dilution 1:1000); rabbit polyclonal anti-human antibodies to GLCL-C and GLCL-R (a gift from Dr Kavanagh, University of Washington, Seattle, WA, dilution 1:1000 for both); a goat polyclonal human antibody to Trx (American Diagnostica, Greenwich, CT, dilution 1:200); and a polyclonal rabbit anti-rat antibody to TrxR (a gift from Dr Arne Holmgren, Karolinska Institutet, Stockholm, Sweden, dilution 1:1000).

The tissue antigens for MnSOD, GLCL-C, GLCL-R, Trx and TrxR were revealed using the Histostain-Plus Kit (Zymed Laboratories Inc, South San Francisco, CA) as described previously [35].

The immunostaining results were evaluated during one session on a multiheaded microscope by three observers (HH, SJ, SP or YS) semiquantitatively by dividing the CA IX and AOE staining reaction into four categories based on the reactivity of the staining taking equally into account both the intensity and extent of the staining: 0 = no immunostaining present; 1 = weak immunostaining, <10% of the tumor tissue immunostained; 2 = moderate immunostaining, 10–50% of the tumor tissue immunostained; 3 = strong immunostaining present, >50% of the tumor tissue immunostained. For the analysis, the four categories were divided into two groups: the CA IX(-) and AOE(-) group contained negatively and weakly stained tumors. The CA IX(+) and AOE(+) group contained tumors showing moderate or strong immunostaining [10].

The immunostaining of AOEs has been described in more detail in our previous study [10]. Briefly, all the AOEs (MnSOD, GLCL-C, GLCL-R, Trx, TrxR) were expressed diffusely in the sections. MnSOD had granular and TrxR perinuclear cytoplasmic immunoreactivity and rest of the AOEs were stained diffusely in the cytoplasm.

The signal for CA IX is both membrane-associated and intracellular in oligodendroglial tumors. CA IX expression mainly showed focal accentuation and was found to be most intense in perinecrotic areas. When necrotic or microvascular proliferation was present, perinecrotic or microvessel staining was commonly seen.

The CA IX and AOE stainings represent adjacent sections in the same area of the tumor.

### Cell proliferation

For the analysis of cell proliferation, a mouse monoclonal antibody MIB-1 recognizing the Ki-67 antigen was used (Immunotech, S.A. Marseille, France) (dilution 1:40). After immunostaining, the tissue sections were counterstained with methyl green. Proliferative activity was reported as a percentage of immunopositive nuclei. Analysis of MIB-1 positive tumor cells was done with a carefully standardized image analysis system (CAS-200™ Software, Becton Dickinson & Co., USA) as described previously [36,37]. Briefly, the analysis was done on areas that were expressing quantitatively the highest number of immunopositive nuclei. Twenty microscopic fields (×400 magnification) were counted along a vertical and horizontal axis perpendicular to each other. Endothelial cells, necrotic and haemorrhagic areas as well as section borders were omitted.

### Apoptotic rate

Apoptosis activity was determined by TUNEL-labeling. The deparaffinized tumor microarray tissue sections were first digested with proteinase K (20 μg/ml) for 15 min. Apoptotic cells were demonstrated using ApopTag In Situ Apoptosis Detection Kit (Oncor, Inc., Gaithersburg, MD, USA) according to the manufacturer's instructions. In the terminal deoxynucleotidyl transferase nick end labeling method, the recommended concentration was reduced eightfold. Direct immunoperoxidase detection of digoxigenin labeled dUTP was followed by counterstaining in methyl green [38]. Staining was analyzed by counting all the tumor cells in the core tissue with an image analysis system (CAS-200™). The obtained scores were reported as a percentage of immunopositive nuclei.

### Analysis of 1p19q

FISH analysis was performed on tumor cell nuclei isolated from paraffin embedded sections. The sections were first deparaffinized with xylene, followed by an ethanol row with decreasing concentrations (100%, 95%, 70%, and 50% for 10 minutes, respectively). After transfer into distilled water, the deparaffinized sections were incubated with 0.1% protease in 0.1 M Tris, 0.07 M NaCl at 37°C for 30 minutes and occasionally agitated. To check cell density, 5 μl of cell suspension was put on a slide and examined under the microscope. After preparing the slides using 10 μl of cell suspension, the slides were air-dried overnight at room temperature and fixed with 4% buffered formaldehyde for 10 minutes. The slides were used immediately or stored in sealed boxes at -70°C. FISH analysis for 1p and 19q were performed with LSI 1p36/LSI 1q25 and LSI19q13/LSI 19p13 probes (Vysis). Hybridization was done according to the protocols of Vysis included in the probe package insert.

Analysis of 1p and 19q loss was done by FISH for 30 most recent tumor samples. In 24 samples there were both 1p and 19q deletions, 1 had only 1p deletion, 1 only 19q deletion, and 4 had no deletions.

### Statistical analysis

The analyses were done by comparing CA IX groups based on the immunostaining reactivity. When stated, CA IX negative group was compared to CA IX positive group.

In survival analysis overall survival was defined from the day of surgery until the death of the patient. The death of patient was considered as an event in the analysis.

Univariate survival analysis was carried out by comparing all the four CA IX categories. In multivariate survival analysis the tumors that had no CA IX immunostaining were compared to those which had weak, moderate or strong staining. This was done to reach the best prognostic cut-off possible.

The tests used for statistical analysis were all part of SPSS 11.0 (SPSS Inc., Chicago, Illinois) for Windows software. Chi-square tests, t-tests, Mann-Whitney tests and Wilcoxon tests were used, as well as multivariate analysis of variance (ANOVA). The log-rank test and Cox multivariate analysis were used for the analysis of prognostic factors. The significance level was set at *p *< 0.05.

### Ethics

The study design was approved by the Ethics committee of Tampere University Hospital and the National Authority for Medicolegal Affairs.

## Results

### Expression of CA IX

**Figure 1:**
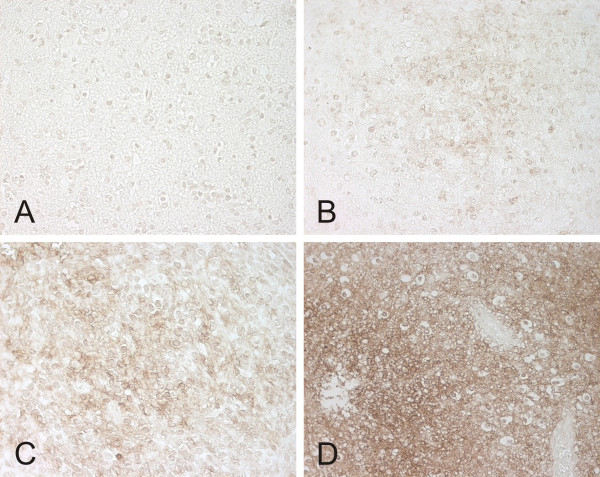
CA IX expression in the four categories (negative (A), weak (B), moderate (C) and strong (D)) in oligodendroglial brain tumor samples. Magnification 400×.

CA IX expression was analyzed in 71 primary and 15 recurrent oligodendroglial tumors. The distribution of immunopositivity for CA IX is shown in Table 1 and examples of immunostained samples in the four categories (negative, weak, moderate, strong) are shown in Figure 1A–D.

The intensity of CA IX immunostaining in different tumor grades is shown in Table 1. There was no significant difference in CA IX expression between grade II and grade III tumors or between primary and recurrent tumors or between pure oligodendroglial tumors and mixed oligoastrocytomas. The age of the patients at the time of the operation did not differ significantly between the CA IX(-) and CA IX(+) groups.

CA IX expression was significantly higher in MnSOD(+) tumors (p = 0.008, chi-square test). On the other hand, CA IX expression was decreased in GLCL-R(+) tumors (p = 0.044, chi-square test). The other AOEs did not reach any statistical association with CA IX. In addition, there was no correlation between CA IX and 1p19q status in our material.

Analyses for cell proliferation and apoptosis were performed and the results were correlated to CA IX expression (CA IX(-) vs. CA IX(+) tumors). Cell proliferation index (MIB-1) was decreased among CA IX(+) tumors. In Ca IX (+) tumors median was 2.9, mean 3.3, SD ± 4.8 vs. median 5.8, mean11.9 and SD ± 13.9 in CA IX(-) tumors (p = 0.015, Mann-Whitney test). No significant difference was seen in apoptotic activity in CA IX(-) vs. CA IX(+) tumors.

### Survival

**Figure 2:**
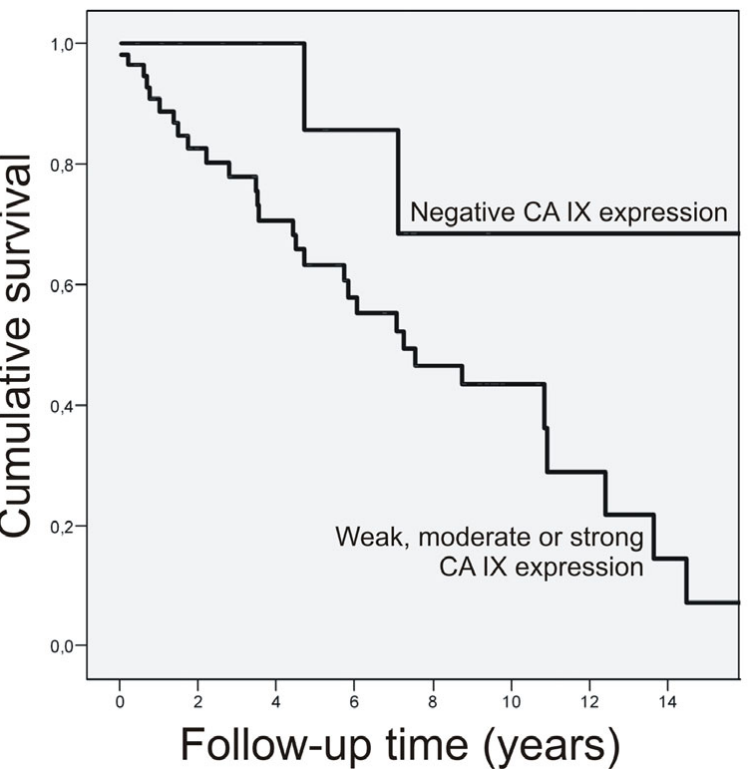
Survival by CA IX in primary oligodendroglial brain tumors.

Survival analysis was confined to the primary cases. When the patients with tumors that had no immunostaining for CA IX were compared in univariate survival analysis with those who had positive immunostaining for CA IX, the negative tumors tended to associate with a better survival rate than their positive counterparts (Figure 2.). However, this difference did not reach statistical significance.

In Cox multivariate analysis the expression of CA IX, patient age and histological component (pure oligodendroglioma vs. mixed oligoastrocytoma) showed independent prognostic significance (p = 0.009, Exp(B) = 7.370; p = 0.003, Exp(B) = 3.422 and p = 0.022, Exp(B) = 0.351). CA IX positivity, older age and astrocytic component predicted poorer outcome. Other clinicopathological features, including proliferation status and 1p 19q status, did not reach statistical significance in association to the prognosis.

## Discussion

We investigated the expression of CA IX in a series of oligodendroglial brain tumors. We found that CA IX is expressed in most of these tumors and that it has an independent prognostic value, predicting a poor outcome. In addition, CA IX(+) tumors had decreased cell proliferation compared to CA IX(-) tumors.

Chromosomal loss of 1p and 19q is a common genotypic change in oligodendrogliomas. It is commonly used as a tool to make more precise decisions concerning the treatment. Recently, it has become clear, however, that defects in single chromosomes, genes, or even the sequential acquisition of mutations, are not the entire story in tumorigenesis, given that many cancer susceptibility genes show a high degree of tissue specificity in their association with neoplastic transformation. Also in oligodendrogliomas and other malignant brain tumors, the acquisition of a complete neoplastic phenotype is caused by the activation of several oncogenes and the inactivation of several growth-suppressor genes, and therefore, a number of target molecules are required to be involved in an optimal treatment strategy. There are several characteristics which are important for such a protein target: The molecule (a) is expressed only in tumor cells, and in the majority of tumor cells; (b) is well accessible on the surface of the cell; (c) is not encoded from a mutated gene, but is derived from a stable chromosomal area; (d) can be targeted with a safe drug or inhibitory antibody; (e) is easy to determine in normal laboratory samples and (f) the costs of diagnostic and therapeutic uses are reasonable.

CA IX is an extremely attractive molecule in cancer research because it has several important properties that make it completely unique. First of all, it is mainly expressed in cancerous tissue. Second, it is expressed in various solid tumor types, including some very common ones (for example breast, lung and cervical carcinomas), which can be quite resistant to chemotherapy and radiotherapy. Third, the CA IX antigen is exposed to the surface of the malignant cell, thus making it accessible for antibodies or cancer drugs. Fourth, the structure of the CA IX molecule is amenable to targeting by drugs and it also has good selectivity. Fifth, CA IX expression can be easily verified from tissues with common laboratory protocols. Sixth, CA inhibitors are already being used in both clinical applications and new trials [33,39] and more effective and safer inhibitors are being developed [40]. Therefore, one could conclude that CA IX indeed exhibits several features that are necessary for a good target molecule in anticancer therapy, and at least for now it is clear that in most cases good cancer treatment includes the best combination of different treatments supporting one another. CA IX and its inhibitors could definitely be considered as part of that kind of treatment strategy in the near future.

CA IX has proved to be a surrogate marker for hypoxia and it has also been found to correlate with prognosis in several cancers (including our present tumor material) [17,26,27,29,41]. CA IX expression could thus be useful in the prognostic assessment of certain tumors. For example, the previous study by Haapasalo et al showed that it has a strong association with tumor grade and poor prognosis in astrocytic brain tumors [28]. CA IX immunopositivity was observed in 78% of astrocytic brain tumors (compared to 80% of oligodendrogliomas in our study). In addition, both in astrocytic brain tumors and in oligodendrogliomas perinecrotic accentuation is quite commonly seen. A study by Preusser et al shows that CA IX has a positive correlation with the presence of necrosis [42]. When necrosis or microvascular proliferation was present, perinecrotic or microvessel staining was commonly seen in our present study, too. This could reflect the severe changes disturbing the tissue (hypoxia, insufficient microvasculature etc.) leading finally to necrosis but also enhancing the expression of CA IX. Although the expression of CA IX is common in both astrocytomas and oligodendrogliomas, it was more clearly associated to tumor malignancy grade in astrocytic brain tumors. However, the unique features of CA IX molecule could be useful when designing improvement of therapeutic interventions for both of these tumor groups.

A recent study by Birner et al in 60 oligodendroglial tumors with 1p aberrations showed that CA IX expression is often accompanied with the expression of hypoxia-inducible factor 1α (HIF-1α) [43]. They concluded that when expressed together, they represent a true tissue hypoxia and not just oncogenic activation, which might otherwise be the case, at least for HIF-1α. It is already known that HIF-1α is one of the key factors regulating cellular O_2 _homeostasis and its activation represent a key step in angiogenesis and adaptation to hypoxia and thus the vitality of the tumor. Overexpression of HIF-1α is shown to be an independent prognostic factor in oligodendroglial tumors and interestingly, it also correlates with the microvessel density in these tumors [44]. They suggested that as tissue hypoxia is known to diminish the efficacy of radiotherapy and thus influence the adjuvant therapy given to the patients, the evaluation of tissue hypoxia using this combination of hypoxia-markers would be helpful for recruitment of patients for individualized therapy strategies, e.g. identification of hypoxic tumors for hyperbaric oxygenation preceding radiotherapy.

In the present study we found that CA IX was expressed more often in MnSOD-positive tumors. In our previous study on AOEs in partly the same subgroup of oligodendroglial brain tumors, MnSOD was associated with the histopathological component (pure oligodendrogliomas vs. mixed astrocytomas), but failed to show a significant correlation with patient survival [10]. The association reported here with CA IX is interesting because CA IX is known to be induced by hypoxia, and MnSOD in turn is induced among other factors by hyperoxia. It seems that CA IX expression and hypoxia are quite consistently associated, while factors that induce MnSOD are quite complex and numerous [7,45]. Another possible explanation for this peculiar association might be that MnSOD and CA IX expression are associated with different phases of tumorigenesis in oligodendrogliomas. MnSOD has been suggested to be a tumor suppressor gene [46,47]. The increased expression of MnSOD may reflect the imbalance during tumorigenesis where cells attempt to control the tumor environment. As the process advances, the environment becomes increasingly hostile for normal cells. Rapidly growing, poorly organized and improperly proliferated cancer cells lead to insufficient microvasculature and low pH, suppressing the growth of normal cells even further, while selectively stimulating the growth of malignant ones. This leads to the activation of hypoxia response elements and consequently induces a number of genes, including the *CA9 *gene, involved in the adaptation to the stress caused by low oxygen. The association between MnSOD and CA IX may thus represent partly overlapping periods of tumorigenesis during which both proteins are present in neoplastic tissue. Furthermore, the basic reaction of MnSOD where reactive oxygen species superoxide O_2_- is converted to H_2_O_2 _by MnSOD is known to affect to HIF-1α, by letting it accumulate into the cytosol. Thus, MnSOD, HIF-1α and CA IX expressions are representing an exciting and complicated continuum.

We also found an association between CA IX and proliferation index (MIB-1). In our material, CA IX(+) tumors had significantly lower proliferation index than their CA IX(-) counterparts. This could reflect the severe hypoxia that is present in the tumor tissue, thus affecting to cells capability to proliferate. A study by Proescholdt et al shows that the relationship between CA IX and proliferation index is quite complicated [48]. They examined the expression of CA IX and CA XII in different kind of brain tumors and also in the normal brain. They found an association between CA IX positivity and increasing proliferation index. However, their material was quite heterogeneous, and oligodendrogliomas were not included at all. Despite of that, it can be said that the correlation between CA IX and proliferation is interesting and should be studied more thoroughly.

## Conclusion

In conclusion, CA IX is expressed in the majority of oligodendroglial tumors and its expression correlates independently with patient survival. It is known to be induced by hypoxia in the tissue, partly by the same elements that induce angiogenesis, and it affects the survival of cancer cells. CA IX seems to play a major role in the pathophysiology of several malignancies, including our material of oligodendroglial tumors, and it may contribute to the abnormal cellular environment of malignant brain tumors by shifting the pH gradient towards higher acidity. Because of its many unique properties, CA IX can be considered a good candidate for a target molecule when designing new anticancer therapies.

## Competing interests

The author(s) declare that they have no competing interests.

## Authors' contributions

SJ participated in the design of the study, light microscopy, 1p19q analyses, analyzed the data and drafted the first version of the manuscript. SP participated in the design of the study, tissue processing, immunohistochemical staining and light microscopy. HB participated to the tissue micro-array blocks from the oligodendroglial tumor samples. MK participated to the 1p19q analyses. AKP participated in the study design and tissue processing. YS participated to the analyses of the AOEs in this study and produced the antibodies for the AOEs. SPas and JP participated in the design of the study and produced and characterized the monoclonal S100P antibody. HH participated in the design of the study, immunohistochemical staining, light microscopy. All authors helped to draft the manuscript. All authors read and approved the final manuscript.

## Pre-publication history

The pre-publication history for this paper can be accessed here:


